# Alteration of hippocampal CA2 plasticity and social memory in adult rats impacted by juvenile stress

**DOI:** 10.1002/hipo.23531

**Published:** 2023-03-25

**Authors:** Radha Raghuraman, Sheeja Navakkode, Sreedharan Sajikumar

**Affiliations:** ^1^ Department of Physiology National University of Singapore Singapore 117593 Singapore; ^2^ Life Sciences Institute Neurobiology Programme Centre for Life Sciences, National University of Singapore Singapore 117456 Singapore; ^3^ Lee Kong Chian School of Medicine Nanyang Technological University Singapore 308232 Singapore; ^4^ Healthy Longevity Translational Research Programme Yong Loo Lin School of Medicine, National University of Singapore Singapore 117456 Singapore; ^5^ Present address: Taub Institute for Research on Alzheimer's Disease and the Aging Brain Columbia University Irving Medical Center New York New York 10032 USA

**Keywords:** area CA2, hippocampus, juvenile stress, long‐term potentiation, social interaction, social memory

## Abstract

The hippocampal CA2 region has received greater attention in recent years due to its fundamental role in social memory and hippocampus‐dependent memory processing. Unlike entorhinal cortical inputs, the Schaffer collateral inputs to CA2 do not support activity‐dependent long‐term potentiation (LTP), which serves as the basis for long‐term memories. This LTP‐resistant zone also expresses genes that restrict plasticity. With the aim of exploring social interaction and sociability in rats that were subjected to juvenile stress, we addressed questions about how the neural circuitry is altered and its effects on social behavior. Although there was induction of LTP in both Schaffer collateral and entorhinal cortical pathways in juvenile‐stressed rats, LTP declined in both pathways after 2–3 h. Moreover, exogenous bath application of substance P, a neuropeptide that resulted in slow onset long‐lasting potentiation in control animals while it failed to induce LTP in juvenile‐stressed rats. Our study reveals that juvenile‐stressed rats show behavioral and cellular abnormalities with a long‐lasting impact in adulthood.

## 1 INTRODUCTION

Hippocampal area CA2 has been shown to play a significant role in social memory formation (Hitti & Siegelbaum, 2014) and spatial encoding (Kay et al., 2016). Given the distinct gene expression patterns and cellular properties in area CA2 compared to areas CA1 and CA3, it is crucial to gain a deeper understanding of how neuronal activity is controlled in this region (Amrita Benoy et al., 2018; Carstens & Dudek, 2019). In contrast to area CA1 and CA3, CA2 pyramidal neurons possess distinct molecular, structural and biophysical characteristics that include an LTP‐resistant phenotype, and many of the genes and proteins known to restrict synaptic plasticity are expressed in this area (Benoy et al., 2018; Carstens & Dudek, 2019; Dasgupta et al., 2017; Zhao et al., 2007). The larger network of inhibitory neurons also wields a tight control over the excitability and plasticity in this region (Leroy et al., 2017; Nasrallah et al., 2015; Piskorowski & Chevaleyre, 2013). CA2 pyramidal neurons drive the hippocampal output onto the area CA1 (Chevaleyre & Siegelbaum, 2010; Kohara et al., 2014; Nasrallah et al., 2019). CA2 neurons project to area CA3 and regulate hippocampal excitability through the recruitment of feedforward inhibitory neurons, and chronic inhibition of CA2‐pyramidal cell synaptic transmission leads to increased excitability of the recurrent CA3 network (Boehringer et al., 2017; Kay et al., 2016). Area CA2 helps establish the dynamic excitatory and inhibitory balance required for proper network function, thus playing a key role as a regulator of network processing in hippocampus (Carstens & Dudek, 2019).

Neurons in CA2 receive long‐range projections from hypothalamic supramammillary (SuM) nucleus (Haglund et al., 1984; Vertes, 1992). SuM projections onto CA2 neurons are active during social novelty, showing that the SuM → CA2 projections convey social novelty signal to hippocampus (Chen et al., 2020). Many of these SuM fibers terminating in CA2 contain substance P (SP) (Borhegyi & Leranth, 1997). SP is one of the abundant neuropeptide found in central nervous system and plays an important role in body's stress response: SP is released in response to stress and activates hypothalamic–pituitary–adrenal (HPA) axis, leading to release of cortisol (Ebner & Singewald, 2006; Iftikhar et al., 2020; Jessop et al., 2000; Mantyh, 2002). Moreover SP and its receptor, neurokinin 1 (NK1) are expressed in brain areas that are involved in the regulation of stress and anxiety responses (Ebner & Singewald, 2006; Mantyh, 2002). The levels of SP and its receptor binding are known to be affected by aversive and stressful stimuli, and an increase in SP concentrations can induce anxiogenic responses (Ebner & Singewald, 2006). We have shown earlier that in the CA2 area, SP induces a protein synthesis‐ and NMDA‐receptor dependent long‐term potentiation. Moreover, SP induced plasticity in Schaffer collateral‐CA2 synapses can transform entorhinal‐CA2 short‐term potentiation into late‐ long‐term potentiation, expressing synaptic tagging and capture (Dasgupta et al., 2017). While SP serves as an useful tool in investigating the effects of antidepressant drugs, the contribution of SP to the understanding of neuropsychiatric disorders and social dysfunctions is unclear (Malek‐Ahmadi, 1992).

Given CA2's role in social behavior in rodents, and that many neuropsychiatric disorders are often associated with social deficits, CA2 dysfunction could contribute to neuropsychiatric disorders in humans. Supporting this notion is the observation that post‐mortem analyses of brains from individuals with schizophrenia reveal alterations in area CA2, including a reduced count of inhibitory neurons in CA2 among patients with schizophrenia and autism compared to control subjects (Benes et al., 1998). In addition, many studies have shown that stress early in life (prenatal, early postnatal or juvenile) increases the risk of developing disorders like depression, anxiety, posttraumatic stress disorder (PTSD), and schizophrenia in adulthood (Anda et al., 2006; Pelcovitz et al., 1994; Turner & Lloyd, 2004).

Compared to the effect of stress in perinatal phase, little is known about juvenile stress and its long‐term consequences on brain and behavior. The juvenile brain is “a brain in transition,” where it undergoes morphological and physiological changes before it matures into adult brain (Romeo & McEwen, 2006). In humans, stress during this phase is associated with anxiety, depression, impulse control disorders and suicide attempts later in life (Weich et al., 2009). Stress in the juvenile phase also remodels cortico‐limbic architecture that modulates emotional behaviors (Eiland et al., 2012). Although the behavioral deficits are observed when animals are stressed in adulthood, they are significantly aggravated when stress is given at juvenile phase, suggesting that juvenile stage might be a critical window in stress susceptibility (Avital & Richter‐Levin, 2005). Thus, it is particularly interesting in light of the finding that area CA2's specific role in social behavior differs depending on the stage of development. For example, CA2 activity is critical for formation of early social memories such as the preference for social familiarity over novelty, encompassing memories that rodents have for their littermates and mothers (Diethorn & Gould, 2022). Therefore, this critical stage of development, during the second postnatal week in mice when CA2 is playing a critical role in the emergence of novelty preference, highlights a possible susceptibility to disruption by insults such as stress.

In addition to the anatomical changes taking place in CA2 during postnatal development (Diethorn & Gould, 2022), a number of molecules regulating synaptic plasticity are changing during this period as well, and they can be modified by early life events like experience. For example, perineuronal nets (PNNs) surrounding CA2 neurons develop postnatally and are changed by environmental enrichment (Carstens et al., 2016; Carstens et al., 2021). Moreover, PNNs are high in area CA2 of the hippocampus of a mouse model of Rett syndrome (RTT) (*Mecp2*‐null) and prematurely restrict plasticity at CA2 synapses in *Mecp2*‐null mice (Carstens et al., 2021). Moreover early‐life enrichment can alter postnatal development of PNNs on CA2 pyramidal neurons indicating that alterations in the rearing environment can affect the development of circuits that have CA2 excitatory synapses (Carstens et al., 2016; Loisy et al., 2023).

Thus, we hypothesized that stress insults during this critical window of CA2 plasticity could result in changes in social behaviors during adulthood. To begin to understand how juvenile stress results in behavioral changes in adulthood, we tested its effects on sociability and social interaction in adult rats. Subsequently, we examined whether synaptic plasticity in CA2 is affected by early life stress.

We found that synaptic responses in hippocampal area CA2 show alterations in LTP in both direct (entorhinal‐cortical, EC) and indirect (Schaffer collateral, SC) pathways after juvenile stress. In addition, juvenile exposure to stress inhibited the potentiation usually observed with the exogenous bath application of SP. Together, these results are suggestive of lasting effects of juvenile stress on CA2 synapses and their capacity for synaptic plasticity changes.

## 2 MATERIALS AND METHODS

### 2.1 Animals

Male Wistar rats that were 3 weeks old were procured from Invivos Pte. Ltd. (Singapore) immediately after the weaning period and subjected to habituation for 3 days in the animal facility. Animals were housed in a room with 12‐h light/ dark cycle with ad libitum access to food and water. When they reached postnatal day (PND) 27, they were subjected to a 3‐day consecutive stress paradigm (see in the following text), and transferred back to their cages at the end of PND27, PND28, PND29 after the respective stress paradigm. They were subjected to slice electrophysiology experiments when they were 5–6 weeks old. A separate cohort of animals of age 5–7 weeks were subjected to sociability and social interaction tests. All the cages were transferred to the behavioral room 30 min prior to the beginning of the first trial. The animal's age and weight were matched, and care was taken so as to not have any prior contact with the animal to be compared. Two control animals were required per experiment, one for session I and one for session II. The same control may have been used between the trials. A total of 82 animals were used for behavioral experiments and 66 animals were used for slice electrophysiology experiments. In all experiments including behavioral and in vitro, we have used male rats for this study, and female rats were excluded to avoid possible hormonal variations that may affect behavior (Autry et al., 2009; Inoue, 2021).

### 2.2 Behavioral experiments

#### 2.2.1 Stress paradigm

The procedure includes three sequential days of exposure to different stressors as mentioned in our previous report (Raghuraman et al., 2022). Briefly, each day involved a different stress protocol, Day 1 (27 PND), forced swim: 10 min forced swim in an opaque circular water tank (diameter: 20 cm; height: 45 cm; water depth: ~38 cm), water temperature 22 ± 2°C. Day 2 (28 PND), elevated platform stress: 100 cm above floor level with a square transparent platform measuring: 21 cm × 21 cm, located in the middle of a small closet‐like room. Rats were subjected to three 30 min trials with intertrial interval of 60 min in the home cage. Day 3 (29 PND), restraint box stress: rats were placed in a metal restraining box that prevents forward‐backward movement and limits side‐to‐side mobility. Rats remained in the restraining box (length: 11.5 cm × breadth: 5.5 cm × height: 4 cm) for 2 h under dim lit condition (Tsoory & Richter‐Levin, 2006). During the stress paradigm, the 82 animals show behavioral signs of stress (freezing immobilization, piloerection, urination, and defecation). The rats were then returned to their home cages, post the completion of stress procedures and were not handled until subjecting them to behavioral paradigm except for weekly weighing and cage sawdust bedding maintenance.

#### 2.2.2 Sociability and social interaction test

The main principle of this test is based on the free choice by a subject animal to spend time near any of the compartments during two experimental sessions, including indirect contact with one or two animals with which it is unfamiliar. To quantitate social tendencies of the experimental animal, the main tasks are to measure: (a) The time spent with a novel conspecific compared to an empty cup and (b) Preference for a novel versus familiar conspecific. “Sociability” in this case is defined as propensity to spend time with another animal, as compared to the time spent alone in an identical but empty chamber. Preference for social novelty is defined as propensity to spend time with a previously unencountered animal rather than with a familiar animal.

#### 2.2.3 Social interaction‐equipment and room set up

**FIGURE 1:**
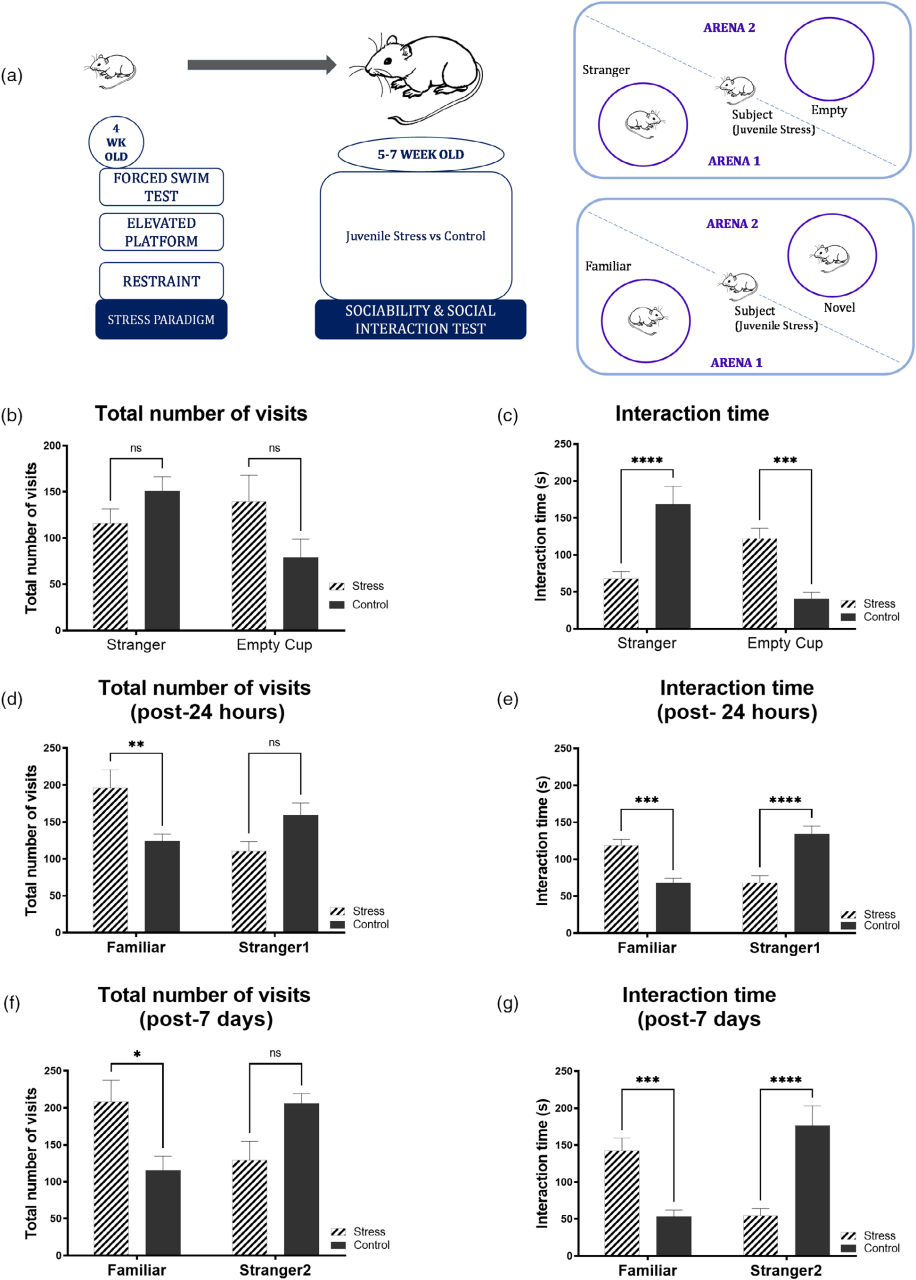
Juvenile stress group shows lower preference to the stranger rats as against control groups that show higher preference for stranger rats in comparison to the empty cups. (a) The schematics of the stress paradigm and the time point where behavioral tests were performed. (b, c) Sociability test; juvenile‐stressed groups spend less time with stranger and more time with empty cup when compared to a control mice. Stressed rats displayed fewer number of visits toward stranger rat as opposed to an empty cup though the difference between stressed and control group was not statistically significant. ((c) Stressed rat toward stranger rat, *p* < .0001, ****p < .0001, *n* = 22); ((c) stressed rat toward empty cup, *p* = .0008, ***p < .001, *n* = 14). The juvenile‐stressed rats showed statistically significant differences compared to the control in terms of total number of visits post 24 h ((d) number of visits, *p* = .00863, **p < .01) with a greater number of events/unit time toward the familiar rat as opposed to a stranger rat. Stressed rats spent a higher time in terms of nose‐point with the familiar rat ((e) nose‐point of stressed rat toward familiar rat, *p* = .0003, ***p < .001, *n* = 14) but a lower amount of time with a stranger rat ((e) nose point of stressed rat toward stranger rat1, ****p < .0001, *n* = 22). The trend was similar in the post‐7‐days interaction test ((f) total number of visits toward familiar rat, *p* = .0170; *p < .05, *p < .05); ((f) total number of visits toward stranger rat 2, *p* = .0887, nonsignificant, *n* = 22).

Behavior testing was performed between 9 a.m. and 6 p.m. General room lighting was 650 lux. After each trial, chambers were cleaned with 70% ethanol and then with Clidox to prevent olfactory cue bias and to ensure proper disinfection, respectively. The arena for each animal is distinguished by specifying corresponding boundaries for each container using the software that gives the read‐out for quantifying the interaction of the subject animal with that of the test. The set‐up for the sociability and social interaction test was a modified version of the three‐chamber paradigm test known as Crawley's sociability and preference for social novelty protocol has been successfully employed to study social affiliation and social memory (Kaidanovich‐Beilin et al., 2011). The arena had two gridded plastic containers to allow for exchange of air between exterior and interior but small enough to prevent direct physical interactions between an animal on the inside with one on the outside (DeVito et al., 2009), The dome of the container was pointed upward (as depicted in Figure 1a), each placed at diagonal ends which was either empty or with a stranger rat underneath it and the test rat was placed in the middle of the arena which did not comprise of any dividing walls.

### 2.3 Social affiliation test (session I)

One of the controls (stranger1) was placed inside a wire containment cup that is located in one side of the arena. The placement of stranger1 in the left or right side of chamber is systematically altered between the trials. Animals were immediately monitored and relevant parameters were recorded such as the duration, number of direct (active) contacts between the subject and the containment cup housing the stranger 1 individually (direct contact between the subject and the containment cup or stretching of the body of the subject is counted as an active contact) and duration and number of entries into each arena. The animal is considered to be in chamber when its head and four paws have entered in to the chamber. The total duration of the above session I is 10 min.

### 2.4 Social novelty preference test (session II)

Second control rat (stranger2) is placed inside an identical wire containment cup in the opposite side chamber (that had been empty during the session I). Same parameters described in the session I were monitored, differentiating the behaviors between the subject animal in the presence of stranger1 compared with stranger2. The total duration of the session II is also 10 min.

### 2.5 Pharmacology

SP (no. 1156; Tocris) was stored at −20°C as a 2.5‐mM stock in deionized water. Before application, the stock solution was diluted to a final concentration of 5 μM in artificial cerebrospinal fluid (aCSF). The preparation procedures, drug application time are similar as demonstrated by earlier studies from our lab (Dasgupta et al., 2017).

### 2.6 Electrophysiology

#### 2.6.1 Slice preparation

All animal procedures were approved by guidelines from the institutional animal care and use committee of the National University of Singapore. Briefly, after anesthetization by using CO_2_, the rats were decapitated and brains were quickly removed, followed by isolation of the right hippocampus. The hippocampus remained cooled in 4°C aCSF with (in mM) 124 NaCl, 2.5 KCl, 2 MgCl_2_, 2 CaCl_2_, 1.25 NaH_2_PO_4_, 26 NaHCO_3_, and 17 D‐glucose equilibrated with 95% O_2_ and 5% CO_2_ (Carbogen) (5). The pH of the aCSF was maintained at 7.3. Transverse slices (400 μm) from the right and left hippocampus were prepared by using a manual tissue chopper, transferred onto a nylon net in an interface chamber (Scientific Systems Design), and incubated at 32°C for 3 h. An aCSF flow rate of 1 mL/min and carbogen consumption of 16 L/h were maintained throughout the experiment. The whole process was carried out within 5 min (Shetty et al., 2015).

#### 2.6.2 Field potential recordings

Three monopolar lacquer‐coated stainless‐steel electrodes (A‐M Systems) were positioned in the hippocampal area CA2. The stimulating electrodes were located in SCs (CA3 → CA2) and cortical fibers (EC → CA2), and the fEPSPs were recorded from the distal dendritic region of the CA2 neurons (Figure 3a). Pathway specificity was tested using the method described in Sajikumar and Korte (2011). The signals were amplified by a differential amplifier and digitized by using a CED 1401 unit (Cambridge Electronic Design). After the incubation period, an input/output curve (stimulus intensity vs. fEPSP slope) was plotted, and the test stimulus intensity was set to obtain a fEPSP slope of 40% of the maximum amplitude. A stable baseline was recorded for at least 30 min before the induction of electrical or SP‐mediated LTP. To induce late‐LTP, STET involving repeated high‐frequency stimulations (three trains of 100 Hz, 100 pulses) was used (Dasgupta et al., 2017).

### 2.7 Statistics

The average values of the slope function of field excitatory postsynaptic potentials (fEPSPs) and excitatory postsynaptic currents (EPSCs) per time point were analyzed by Wilcoxon signed‐rank test when compared within the same group (before and after induction of synaptic plasticity). The Mann–Whitney *U* test was applied when compared between groups. Statistical significance for behavioral paradigm was evaluated by two‐way ANOVA (mixed model 2‐way ANOVA) and the *t*‐test. Results were considered as significant at *p* < .05. The statistical analyses were performed using the Prism software (GraphPad9).

## 3 RESULTS

### 3.1 Sociability and social interaction test in adult rats subjected to juvenile stress

The first session of the test presents the estimation of social motivation levels and social affiliation of the subject rat. The stranger rat that was enclosed in a circular wire cup that allows nose contact yet prevented fighting between the animals in this part of the test ensured that social approaches are initiated by the subject rat and is purely investigatory with no direct physical contact. Given that the rat has a choice to spend as much time as desired around the containment cup containing the stranger rat or to circumvent any contact by heading to the arena that has the empty cup

Figure 1a represents the timeline and scheme of the behavioral paradigm. To start, we compared the performance of control rats to that of stressed rats in a sociability test, which assesses a subject rat's normal preference for a chamber containing a stranger versus an empty chamber. The results showed that stress did not affect sociability in terms of the total number of visits to the stranger and empty cup. The total number of visits represented is the total number of visits in the recorded time period of 600 s. Both the control group and the juvenile‐stressed group displayed a comparable preference for the compartment containing the stranger rat and an empty cup. There were no significant differences in the total number of visits to stranger or empty cup when compared between stressed and control rats (Figure 1b. *p* = .4163 for stress vs. control for stranger rat and *p* = .0942 for stress vs. control for empty cup)

In contrast, the juvenile stress group showed a significant decrease in the number of nose‐points (which were taken as a measure of interaction time in seconds) with the stranger, compared to the control rat. The stressed rats spent significantly less time with a stranger rat compared to the control (Figure 1c, *p* < .0001, ****p < .0001, *n* = 22). Evidently, the stressed rat spent significantly more time with an empty cup compared to the control rat (Figure 1c, *p* = .0008, ***p < .001, *n* = 14). This inclination toward empty cup in comparison to that of the stranger rat, demonstrates an impaired sociability in these juvenile‐stressed animals. Moreover, the difference in score with respect to total number of visits and interaction time (time spent exploring the strange rat minus time spent exploring the empty cup) of the stressed group was significantly less than that of the control group. Difference in score for stranger minus empty cup of the stressed group versus control group is given by Figure S1A—*p* = .0007, ***p < .001 for total number of visits, and difference in score for stranger minus empty cup for the interaction time given by Figure S1B—****p < .0001.

The second session of the test was designed to evaluate social novelty and memory, 24 h after the sociability test, the rat was given a free choice between two options: the first candidate it encountered (now familiar), and a new, never‐before‐met stranger rat (stranger1). A control animal, with its intact social memory and preference for novel experiences, would be more likely to spend time with the newly encountered rat (stranger1). This test measured social recognition by comparing the increased time a subject rat spent interacting with the stranger1, unrelated rat to the time it spent interacting with a familiar rat.

Contrary to their sociability, juvenile‐stressed rats subjected to stress showed a significant impairment in social recognition as revealed by the social novelty test. Interestingly, the juvenile rats subjected to stress did not show a preference for the newly introduced rat (stranger1) over the now familiar rat (Figure 1d,e). The juvenile‐stressed rats showed statistically significant differences compared to the control in terms of total number of visits (Figure 1d, *p* = .0086, **p < .01) with a higher events/unit time toward the familiar rat as opposed to a stranger rat. This was also reflected in the preference to spend more time near the container with the familiar rat given by nose‐point as shown by the interaction time (Figure 1e, *p* = .0003, ***p < .001) and a lower number of nose‐point with a stranger rat (Figure 1e, ****p < .0001). The difference in score with respect to total number of visits and interaction time (time spent exploring the familiar rat minus time spent exploring the stranger rat) of the stressed group was significantly higher than that of the control group. Difference in scores for familiar minus stranger1 rat for total number of visits is given in Figure S1C, *p* = .0020, **p ≤ .001 for the difference in score for total number of visits and Figure S1D, ****p < .0001 for difference in score (familiar minus stranger1) for interaction time.

A similar behavior was observed in the social interaction test conducted 7 days after the sociability test, where the juvenile‐stressed rats showed statistically significant values for both the total number of visits and duration of their interaction with the more familiar candidate, as opposed to another novel stranger rat (stranger2) (Figure 1f, total number of visits, *p* = .0318, *p < .05, visits toward familiar rat).The stressed rats show significantly higher preference in terms of total number of visits toward the familiar rat. However, this is not reflected in terms of the nose‐point/interaction time, where they show a lower preference to the stranger rat 2 although it is not statistically significant (Figure 1f, *p* = .0887, total number of visits toward stranger rat 2).

The juvenile‐stressed rats show a significantly higher nose‐point/interaction time compared to the control toward familiar rat (Figure 1g, *p* = .0008, ***p < .001, Nose point of stressed rat toward familiar rat) and a significantly lower nose‐point/interaction time with a stranger rat 2 (Figure 1g, ****p < .0001, Nose point of stressed rat toward stranger rat). Difference in score between familiar minus stranger 2 rat with regards to the total number of visits is given by (Figure S1E, *p* = .0296, *p ≤ .05 for difference in score (familiar minus stranger2) in total number of visits and Figure S1F, ****p ≤ .0001 for the difference in score between (familiar minus stranger2) in interaction time). This atypical behavior of juvenile rats subjected to stress in this test is suggestive of decreased social motivation and novelty.

### 3.2 Impairments in sociability and social novelty in juvenile‐stressed animals by looking at the delay time in latency‐to‐first

**FIGURE 2:**
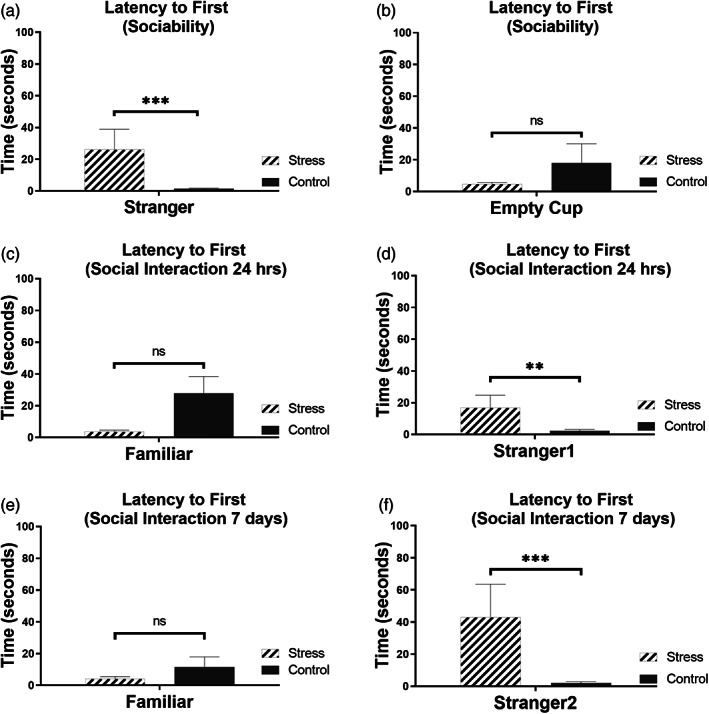
Latency‐to‐first for stress group shows higher delay for never‐before‐met strangers as against familiar subjects. (a, b) The sociability of stressed group in comparison with the control group. (a) The comparison of stress versus control when subjected to stranger rat which is introduced for the very first time. The stressed rat shows a significant increase in the latency‐to‐first (in seconds) to the stranger rat ((a) latency‐to‐first, *p* = .0004489, ***p < .0015). The stressed rat shows relatively lower time in establishing contact with an empty cup compared to the control group ((b) latency‐to‐first with empty cup, *p* = .3914, nonsignificant). (c, d) Latency‐to‐first post 24 h when subjected to a familiar versus never‐before‐met stranger rat. The stressed rat shows a lower time to establish contact with a familiar rat but significantly more amount of time to establish contact with the stranger rat1 ((c) *p* = .0781, nonsignificant; (d) *p* = .0038, **p < .01). (e, f) Latency‐to‐first post‐7‐days of interaction with a novel conspecific wherein (e) shows a lower value in terms of seconds for the stressed rat to establish contact with a familiar rat as opposed to a longer amount of time to establish contact with a never‐before‐met conspecific, named here as stranger rat 2 ((e) *p* = .1663, nonsignificantly lower; (f), *p* = .0002, ***p < .001).

Latency‐to‐first is a parameter that measures the time (measured here in seconds) taken by the animal to establish first contact with the containers (with either the stranger‐rat or the empty container) placed in either of the respective arena. As is evident from the latency‐to‐first values of the stressed rats show a longer time to establish contact with a conspecific that it has never met before showing a significantly higher difference in time when compared to a control rat (Figure 2a, latency‐to‐first, *p* = .0004, ***p < .001). The stressed rats show comparatively less time to establish contact with the empty container as against control rats showing smaller delay toward a stranger rat although there was no statistical significance. Thus latency‐to‐first for sociability test of juvenile‐stressed rats suggests that a stressed animal displays larger delays in the time taken to establish contact with a never‐before‐met stranger as compared to a control rat (Figure 2a,b).

In the second session, when the rat was given a choice to choose between the candidate that it was made to encounter first, already‐investigated (which is now familiar) and a novel never‐before‐met stranger (stanger1), the stressed rats showed more preference toward the familiar rat (Figure 2c) compared with control rats which show more preference to the never‐before met stranger1 rats; the stressed rats showed a significantly lower preference to the stranger rat 1 when compared to the control group (Figure 2d), Latency‐to‐first, *p* = .0038, **p < .01. Thus latency‐to‐first for social interaction 24 h post sociability test in stress group shows higher delay in time in establishing first contact with their never‐before met stranger1 rats compared to the control group that shows lesser delay in time in establishing first contact with their never‐before‐met stranger1 rats.

A similar behavior is seen in the case of social interaction post 7 days of the sociability test wherein juvenile‐stressed rats show significantly lower values for latency–to‐first values for their interaction with a more familiar candidate (Figure 2e) as against another never‐before met candidate, stranger2 (Figure 2e) as against control rats that show more preference to the novel subjects instead of familiar ones (Figure 2f, latency‐to‐first, *p* = .0002, ***p < .001). Thus latency‐to‐first for social interaction at 7 days post sociability test in stress group also shows higher delay in time in establishing first contact with their never‐before met stranger rats compared to the control group. Our results show that rats which are subjected to juvenile stress show impairments in sociability and reduced social interaction toward novel subjects as compared to familiar candidates.

### 3.3 Juvenile stress alters long‐term potentiation in area CA2 synapses

**FIGURE 3:**
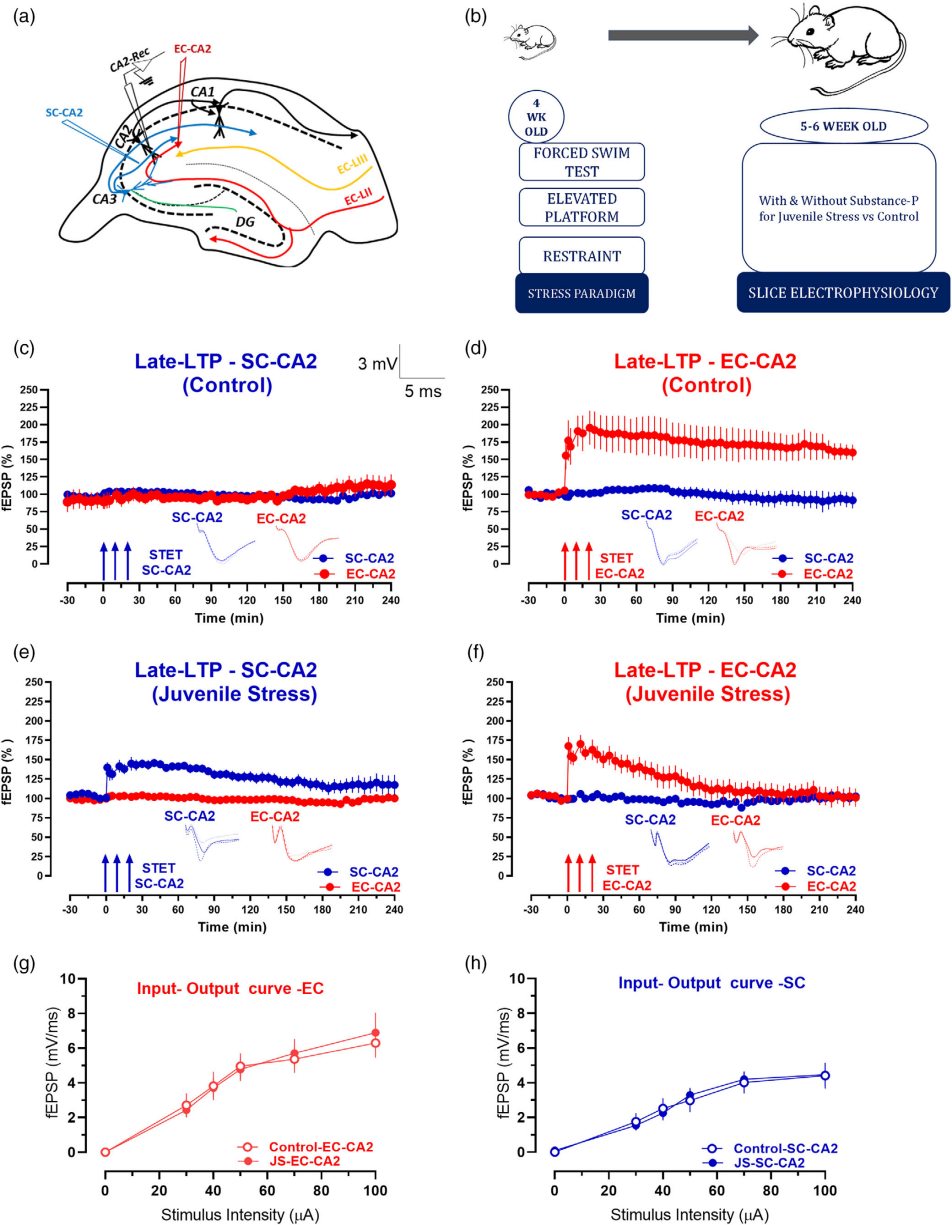
(a) Scheme shows the location of two stimulating electrodes in SC‐CA2 and EC‐CA2 and a recording electrode to record the fEPSPs within the hippocampal CA2 region. (b) Scheme depicts the schema of stress paradigm and the time point where slice electrophysiology experiments were performed (c) STET at SC‐CA2 did not lead to the expression of LTP (blue circles, *n* = 9). The EC‐CA2 inputs showed stable potentials throughout the recording period (red circles). (d) STET‐induced long‐lasting input‐specific LTP in EC‐CA2 inputs (red circles) with stable control potentials in SC‐CA2 (blue circles; *n* = 5). (e) Application of STET in the SC‐CA2 (juvenile‐stressed rats) input showed an early‐LTP lasting until 60 min but declined to baseline levels starting from 65 min onward until the end of the recording of 240 min (blue circles, *n* = 9). (f) Similarly, application of STET in the EC‐CA2 showed only an early‐LTP until 70 min post which it began declining till the end of recording of up to 240 min (red circles, *n* = 12). The baseline responses from the SC‐CA2 input were unaffected throughout the recording period in both (e, f). “*n*” represented the number of slices. (g, h) The input/output (IO) curves obtained by plotting the increased slope values of fEPSPs corresponding to the enhanced stimulus intensities from EC and SC of CA2 region of hippocampus. Representative fEPSPs, 15 min before (dotted line), 30 min after (hatched line), and 240 min after (continuous line) STET are depicted in arrows. Calibration bars are 3 mV/5 ms.

Next, we were curious to know if the behavioral alterations that we observed in juvenile stress were also reflected at cellular level in CA2. Figure 3a,b depicts the scheme of the hippocampal slice and the timeline of the electrophysiology experiments after stress paradigm, respectively. It has been demonstrated previously that the SC synapses in area CA2 are resistant to the induction of activity‐dependent plasticity such as LTP, whereas EC synapses are not (Chevaleyre & Siegelbaum, 2010; Zhao et al., 2007). The same has also been shown previously from our lab (Dasgupta et al., 2017). We repeated these experiments and showed that strong tetanic stimulation (STET) failed to induce LTP at SC synapses, while STET in EC synapses induces a long‐lasting LTP (Figure 3c,d). Interestingly, in the case of hippocampal slices from rats that were subjected to juvenile stress, both SC‐CA2 as well as EC‐CA2 synapses showed induction of LTP but failed to express late‐LTP (Figure 3e,f). SC‐CA2 LTP showed potentiation until 60 min (141.3 ± 4.973; Wilcoxon test, *p* = .0078, *n* = 9). From 65 min onward, the potentiation was not significant (100.7 ± 1.399; Wilcoxon test, *p* = .4258, *n* = 9) and began declining until the end of the recording of 240 min (111.8 ± 12.64; Wilcoxon test, *p* = .7344, *n* = 9, Figure 3e). Similarly, the EC‐CA2 showed potentiation until 70 min (148.7 ± 14.95; *p* = .0342, *n* = 12) after which time the potentiation began declining (129.0 ± 15.28; *p* = .1514, *n* = 12) until 240 min, the end of the recording (101.0 ± 11.81; *p* = .7334, *n* = 12, Figure 3f). In all cases, the control baseline remained stable throughout the recording period of 240 min (Wilcoxon test, *p* > .05). Figure 3g,h shows curves of the fEPSP slopes plotted against stimulus intensity (input/output—IO) curves from EC and SC of CA2 region of hippocampus. No significant changes were observed between control EC‐CA2 (red, open circles) and JS‐EC‐CA2 (red, filled circles) (Figure 3g) and also between control SC‐CA2 (blue, open circles) and JS‐SC‐CA2 (blue, filled circles) (Figure 3h).

### 3.4 SP failed to induce LTP in area CA2 in juvenile‐stressed rats

**FIGURE 4:**
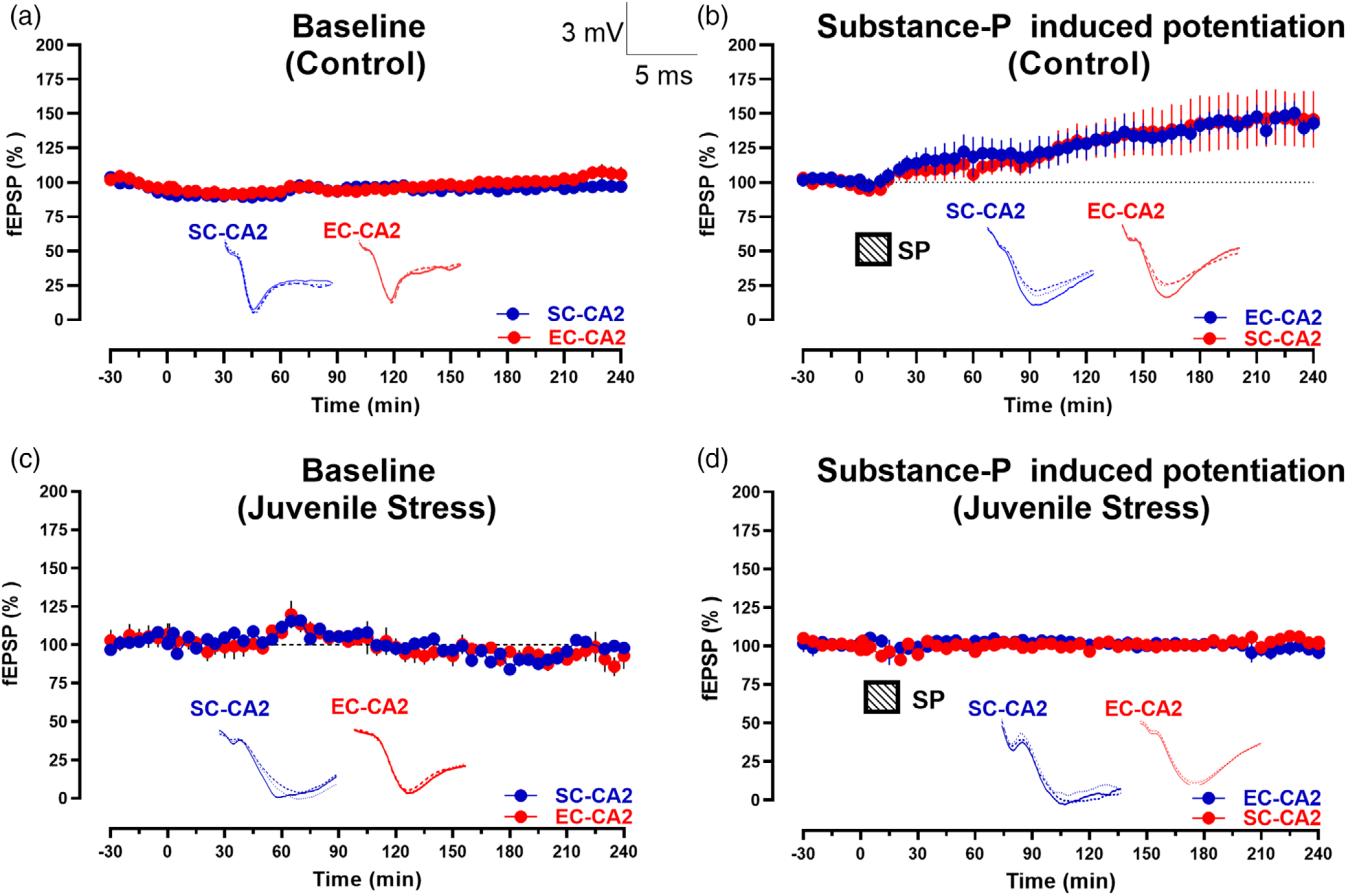
SP application does not result in potentiation in hippocampal slices from the juvenile‐stressed rats. (a) Control slices showing the stability of recordings with no SP application in the CA2 region (*n* = 7). (b) Application of SP for a total duration of 15 min after recording a stable baseline for about 30 min, showed slow‐onset potentiation of fEPSPs in both studied synaptic inputs, EC‐CA2 (blue circles) and SC‐CA2 (red circles) (*n* = 5). (c) Control hippocampal slices from juvenile‐stressed rats showing no potentiation of SC‐CA2 (blue circles) or EC‐CA2 (red circles) with no application of SP, *n* = 6. (d) Application of SP for a total duration of 15 min post the recording of a stable baseline for 30 min showed no potentiation in the juvenile‐stressed hippocampal slices in either EC‐CA2 (red circles) or SC‐CA2 synapses (blue circles) (*n* = 9).

It can be recognized from our data that neuropeptide SP shows a slow onset potentiation leading to a late‐phase LTP in both Schaffer collateral and entorhinal cortical (EC) pathways in hippocampal CA2 in control rats (Figure 4b) in comparison to the control slice sans SP application (Figure 4a). However, in the case of juvenile‐stressed rats, contrary to the above findings, an exogenous bath application of SP did not result in slow‐onset long‐term potentiation (Figure 4c,d). There was no significant difference in the mean fEPSP slope values between the SC‐CA2 and EC‐CA2 synapses compared to the controls (Mann–Whitney *U* test, *p* > .05, *n* = 9). Similarly, there was no significant difference in the mean fEPSP slope values between both the groups in the control (with a lack of exogenous SP bath application) in either of the inputs (Mann–Whitney *U* test, *p* > .05, *n* = 6).

## 4 DISCUSSION

Many neuropsychiatric disorders develop in early childhood. Early life stressful events have considerable implications in adult life and can increase the risk for psychiatric disorders (Targum & Nemeroff, 2019). During these periods of particularly high rates of synaptic remodeling in the brain, experiences can shape the neural plasticity and therefore any adverse effects can have long‐lasting implications. Although significant progress has been made in the understanding of how early/juvenile stress affects behavior, a mechanistic linkage between the juvenile stress and social behavior is still missing. The CA2 region contains receptors for several neuromodulators associated with various aspects of social behavior, suggesting that it plays a crucial role in processing social information in the brain. Hitti and Seigelbaum's studies showing genetic inactivation of CA2 neurons leads to loss of social memory revealed CA2 as a hub of sociocognitive memory processing. As a number of neuropsychiatric diseases are associated with alteration of social phenotypes, it raises the possibility that CA2 dysfunction could contribute to behavioral changes (Hitti & Siegelbaum, 2014). Apart from genetic silencing, chemogenetic and optogenetic silencing of dorsal CA2 also result in deficits in social memory, including encoding, consolidation and recall (Meira et al., 2018). Using genetic and pharmacological approaches Raam et al. showed that oxytocin receptors in the anterior dentate gyrus and anterior CA2/CA3 of mice are necessary for discrimination of social stimuli (Raam et al., 2017). Further evidence supporting the role of CA2 in social memory comes from the group of Dudek SM showing that CA2 neurons encode novel and social information by modifying existing spatial representations (Alexander et al., 2016). Okuyama et al. showed that ventral CA1 (vCA1) and its projections to Nucleus accumbens (NAc) shell play a necessary and sufficient role in social memory (Okuyama et al., 2016). As dorsal CA2 (dCA2) neurons have longitudinal projections to ventral CA1, it is possible that the dCA2‐vCA1d‐NAc circuit composes the engram cell ensemble pathway for social memory (Tonegawa et al., 2015). In addition, Piskorowski et al showed an age‐dependent alteration in CA2 in *Df(16)A* ^*+/−*^ mouse model of the 22q11.2 microdeletion, which is a genetic risk factor for developing several neuropsychiatric disorders (Piskorowski et al., 2016). These effects are observed post maturity until the stage of adulthood, paralleling the onset of disease in humans. These studies highlight the significance of hippocampal area CA2 and the associated circuitry yet to be largely explored that could potentially be involved in social memory.

The results of our present study exemplify that exposure to juvenile stress can modulate the behavior in adulthood. The first goal of our study was to characterize various social behavioral phenotypes in adulthood, impacted by juvenile stress. We observed that juvenile‐stressed rats exhibited decreased sociability, which is the propensity to spend time with another rat, compared to time spent with an empty cup. The control rat was observed to spend significantly more time with the stranger rat as against empty cup that is indicative of sociability or social motivation. In contrast, the rat that was subjected to juvenile stress spent more time with empty cup than the stranger rat, showing an impaired sociability in juvenile stress model. The finding that juvenile stress has decreased sociability is consistent with studies in humans and other animals that showed that early life stress is associated with a number of behavioral abnormalities in addition (Toth et al., 2008; Watt et al., 2017). Thus, the physiological systems regulating social behavior are developing in a way that makes them highly vulnerable stress experienced during early life. These findings collectively highlight a crucial time period, known as the “critical window,” or “sensitive period” during which early life stress has the ability to alter the developmental programming. The effects of early stress also depend on the timing of exposure and genetic factors (Sandi & Haller, 2015).

In the social novelty and social memory test, when the rat was given a free choice between familiar and never‐before‐met stranger, a normal rat would recall its previous contact with stranger rat, by the virtue of its intact social memory and prefers social novelty. Thus “preference for social novelty” is the propensity to spend more time with a novel rat than a familiar rat. Our data shows that one repercussion of juvenile stress is a decrease in social motivation and social novelty. Our studies are also consistent with other studies that showed changes in adult social behaviors without affecting cognitive functions in juvenile stress condition (Toth et al., 2008). This shows that stress in early life poses increased risk for depression and other neuropsychiatric diseases.

As a number of neuropsychiatric disorders are linked to altered social phenotypes, our findings raise the probability that CA2 dysfunction may contribute to the observed social behavioral alterations. This is reinforced by the findings of a lower number of CA2 inhibitory neurons in patients with schizophrenia and bipolar disorder (Benes et al., 1998). It is known that inhibitory synaptic transmission in the hippocampal CA2 neurons are sensitive to changes in environment and that environmental enrichment (EE) reduce GABA transmission (Loisy et al., 2023). Moreover, 24 h of social isolation in an EE reversed the EE‐induced changes in synaptic transmission which shows that hippocampal area CA2 neurons are particularly vulnerability to social isolation stress (Loisy et al., 2023). This is also supported by the studies from Carstens et al showing that circuits containing CA2 excitatory synapses are sensitive to manipulations of the rearing environment where they showed that postnatal development of PNNs on CA2 pyramidal neurons is modified by early‐life enrichment (Carstens et al., 2016). We showed that SC‐CA2, which is a plasticity‐restricted area, expressed early‐LTP in juvenile‐stressed rats, while EC‐CA2 showed impaired LTP. This alteration of plasticity observed in juvenile stress rats in the hippocampal area CA2, can be tied in to the studies showing that ventral hippocampal slices from stressed rats expressed larger LTP than that produced in the dorsal hippocampus (Maggio & Segal, 2011). Normally the ventral hippocampus expresses lower magnitude LTP compared with the dorsal hippocampus (Trompoukis & Papatheodoropoulos, 2020). The alteration of LTP in CA1 ventral hippocampus due to juvenile stress is known to increase the expression of beta1‐adrenergic receptors in the ventral but not in the dorsal hippocampus (Grigoryan et al., 2015). Thus, a change in the expression of neuromodulatory inputs due to juvenile stress could alter the plasticity in the area CA2, which is important for social memory. Juvenile stress might also bring about synaptic competition, possibly due to lack of necessary PRPs, which leads to impairment of late phase of LTP (Sajikumar et al., 2014).

We have shown earlier that SP induces long‐term potentiation and facilitates synaptic tagging and capture (Dasgupta et al., 2017). Although SP is known to be involved in the regulation of stress and anxiety (Ebner & Singewald, 2006), little is known about the mechanisms by which it regulates plasticity changes in hippocampal area CA2. Stressful and aversive stimuli have repeatedly been demonstrated to alter both the brain content SP levels and the binding of NK1 receptors (Ebner & Singewald, 2006). In addition, it may provide also valuable insights into the study of the effects of antidepressant and electroconvulsive therapy (Malek‐Ahmadi, 1992).

SP expression has been observed to change and its levels can be influenced by various factors, including hormonal and environmental factors (Ebner et al., 2004; Zalecki, 2019). Thus, juvenile stress might alter the levels of SP in CA2 at this critical time window to have long term implications in behavior. Additionally, studies in animals have suggested that SP levels may be important for the development of the nervous system and the regulation of certain behaviors (Ebner et al., 2004). However, more research is needed to fully understand the developmental regulation of SP.

Although juvenile‐stressed rats expressed an early form of LTP in SC and EC inputs, SP, which is known to induce NMDA‐receptor and protein synthesis‐dependent slow onset potentiation in both SC and EC inputs of CA2 (Dasgupta et al., 2017), failed to induce potentiation in juvenile‐stressed rats. It is also known that the delta opioid receptors (DOR) in the inhibitory interneurons decreases the inhibitory transmission without affecting the excitatory transmission from CA2. It was also shown that increase in action potential firing after HFS is dependent on DOR, thus a presynaptic inhibitory plasticity might allow the CA2 output to increase by decreasing the inhibition with no change in the excitation, which might account for the CA2 plasticity (Nasrallah et al., 2015). Moreover, SP has been associated with axonal terminal to inhibitory neurons which might explain the slow onset LTP seen in the control animals subserved by the disinhibition effect. One possible mechanism for this loss of SP‐induced potentiation in the juvenile‐stressed animals might be that the endogenous release of SP as a result of stress, and a subsequent LTP, occludes the induction of LTP as in the case of chronic pain (Zieglgansberger, 2019). This can be due to saturation of synaptic strengthening, or a competition for limited resources or interference in signaling pathways. Our work is suggestive of juvenile stress condition inducing behavioral metaplasticity in the hippocampal area CA2 that may interfere with mechanisms behind social memory and social interaction. Our findings that juvenile‐stressed rats show reduced social novelty is also supported by findings that social interactions with both novel and familiar animals are known to rearrange place maps in CA2 (Alexander et al., 2016), but not CA1.

We have shown recently that cholinergic receptor activation induces an LTD of synaptic transmission at EC and SC‐CA2 synapses. This priming of synapses through neuromodulation by acetylcholine‐mediated LTD displays a bidirectional metaplastic switch to LTP on a futuristic timescale (Benoy et al., 2021). On that note, it will be worthwhile to explore whether SP mediated social memory after juvenile stress also emerges through similar mechanisms. Recent studies from Dudek group have shown that deletion of mineralocorticoid receptors (MRs), either at the embryonic or postnatal stage, impedes development inputs from SuM into CA2 and DG in addition to causing a decline in the expression of proteins that help characterize CA2 neurons (McCann et al., 2021). These findings are in support with our current work that corroborates the effects of stress on CA2 and SuM connectivity (McCann et al., 2021). Whether it is the MRs that are indeed depleted/impaired in the case of juvenile‐stressed rats requires further investigation.

The cellular mechanisms underlying social memory in juvenile stress animal model will help us to explore potential brain areas or circuits of neuropsychiatric disorders. The juvenile stress condition represents an excellent model system to investigate developmental conditions, given the significant effects it has on adult social behavior. This will, in addition, help highlight the importance of timely interventions to prevent the adverse effects by providing favorable conditions like environmental enrichment, novelty etc. at different points in life.

## FUNDING INFORMATION

This work was supported by Ministry of Health (MOH‐000641‐00), Ministry of Education Academic Research Fund Tier 3 (MOE2017‐T3‐1‐002), NUHS Seed Fund (NUHSRO/2020/145/RO5+6/Seed‐Sep/05) and NUSMED‐FOS Joint Research Programme (NUHSRO/2018/075/NUSMed‐FoS/01) to Sreedharan Sajikumar. Radha Raghuraman is supported by NUS Research Scholarship, National University of Singapore.

## Supporting information


**Supplementary Figure S1.** The figure shows the difference in score in sociability with respect to total number of visits (A) and interaction time (B) (difference in score for stranger minus empty cup of the stressed group vs control group given by Figure S1. A, *P* = 0.0007, ***P < 0.001 for total number of visits, and difference in score for stranger minus empty cup for the interaction time given by Figure S1. B, ****P < 0.0001). S Figure 1c,d shows the difference in score in social novelty with respect to total number of visits (C) and interaction time (D) (time spent exploring the familiar rat minus time spent exploring the stranger rat) of the vs control group after 24 hours. (Figure S1.C, P = 0.0020, **P < =0.001 for the difference in score (familiar minus stranger1) for total number of visits and Figure S1. D, ****P < 0.0001 for difference in score (familiar minus stranger1) for interaction time). S Figure 1e and 1F shows the difference in score in social novelty with respect to total number of visits (E) and interaction time (F) (time spent exploring the familiar rat minus time spent exploring the stranger rat) of the vs control group after 7 days. (Figure S1. E, *P* = 0.0296, *P < =0.05 for difference in score (familiar minus stranger2) in total number of visits and S1. F, ****P < =0.0001 for the difference in score between (familiar minus stranger2) in interaction time).

## Data Availability

The data that support the findings of this study are available from the corresponding author upon reasonable request.
