# Supplementary figures and images for: Alteration of hippocampal CA2 plasticity and social memory in adult rats impacted by juvenile stress
Source: Hippocampus. 2023 Mar 25;33(6):745–58. doi: 10.1002/hipo.23531 (PMC10946601; doi:10.1002/hipo.23531)

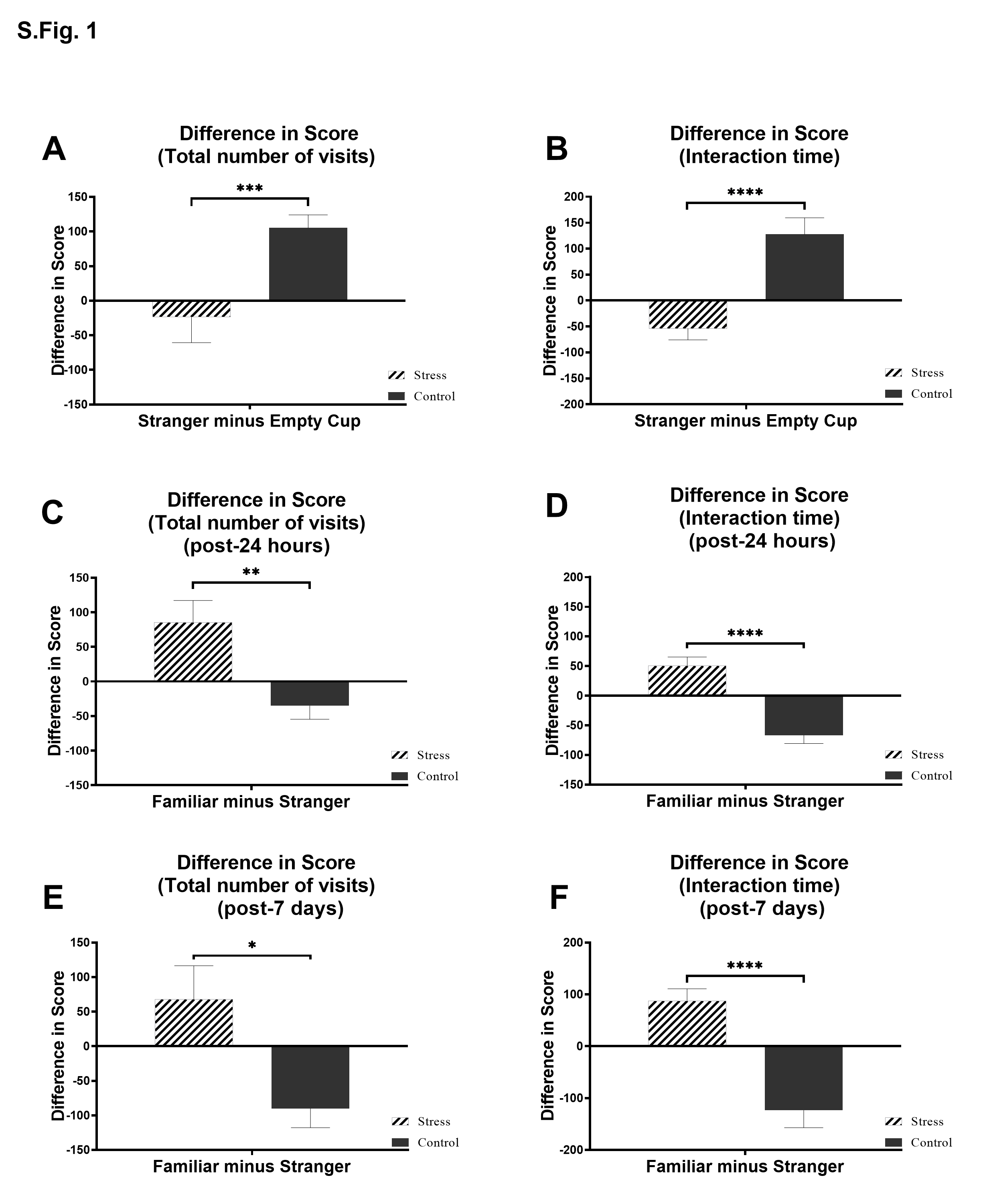

Supplement: Supplementary file 1 — Supplementary Figure S1. The figure shows the difference in score in sociability with respect to total number of visits (A) and interaction time (B) (difference in score for stranger minus empty cup of the stressed group vs control group given by Figure S1. A, P = 0.0007, ***P < 0.001 for total number of visits, and difference in score for stranger minus empty cup for the interaction time given by Figure S1. B, ****P < 0.0001). S Figure 1c,d shows the difference in score in social novelty with respect to total number of visits (C) and interaction time (D) (time spent exploring the familiar rat minus time spent exploring the stranger rat) of the vs control group after 24 hours. (Figure S1.C, P = 0.0020, **P < =0.001 for the difference in score (familiar minus stranger1) for total number of visits and Figure S1. D, ****P < 0.0001 for difference in score (familiar minus stranger1) for interaction time). S Figure 1e and 1F shows the difference in score in social novelty with respect to total number of visits (E) and interaction time (F) (time spent exploring the familiar rat minus time spent exploring the stranger rat) of the vs control group after 7 days. (Figure S1. E, P = 0.0296, *P < =0.05 for difference in score (familiar minus stranger2) in total number of visits and S1. F, ****P < =0.0001 for the difference in score between (familiar minus stranger2) in interaction time). [file HIPO-33-745-s001.tif]
